# Readmissions of adults within three age groups following hospitalization for pneumonia: Analysis from the Nationwide Readmissions Database

**DOI:** 10.1371/journal.pone.0203375

**Published:** 2018-09-13

**Authors:** Snigdha Jain, Rohan Khera, Eric M. Mortensen, Jonathan C. Weissler

**Affiliations:** 1 Division of Pulmonary and Critical Care Medicine, Department of Internal Medicine, UT Southwestern Medical Center, Dallas, TX, United States of America; 2 Division of Cardiology, Department of Internal Medicine, UT Southwestern Medical Center, Dallas, TX, United States of America; 3 Division of General Internal Medicine, University of Connecticut Health Center, Farmington, CT, United States of America; Duke-NUS Medical School, SINGAPORE

## Abstract

**Background:**

While 30-day readmissions following hospitalization for pneumonia have been well-studied in the elderly, their burden in young adults remains poorly understood.

**Objective:**

To study patterns of readmissions following hospitalization for pneumonia across age groups and insurance payers.

**Methods:**

In the Nationwide Readmission Database for the years 2013 and 2014 we identified all adults (≥18 years) discharged alive after a hospitalization with the primary diagnosis of pneumonia, and examined rates of readmissions within 30-days of discharge. Using covariates included in the Center for Medicare & Medicaid Services risk-adjustment model for pneumonia readmissions in a multivariable regression model for survey data, we identified predictors of 30-day readmission.

**Results:**

We identified 629,939 index pneumonia hospitalizations with a weighted estimate of 1,472,069 nationally. Overall, 16.2% of patients were readmitted within 30 days of their hospitalization for pneumonia, with 30-day readmission rates of 12.4% in the 18–44 year age-group, 16.1% in the 45–64 year age-group, and 16.7% in the ≥65-year age-group. In risk-adjusted analyses, compared with elderly, middle-aged adults were more likely to be readmitted (risk-adjusted OR 1.05, 95% CI 1.03–1.07). Mean cost per readmission was also highest for this age group at $15,976.

**Conclusion:**

Middle-aged adults experience substantial rates of 30-day readmission that are comparable to those over 65 years of age, with a higher cost per readmission event. Future efforts are needed to identify potential interventions to alleviate the high burden of pneumonia readmissions in middle-aged adults.

## Introduction

Pneumonia is the most common medical cause of hospitalization in the United States.[1] Among Medicare beneficiaries, one in five patients discharged after a hospital stay for pneumonia is readmitted within 30 days.[2] While considerable effort has been targeted towards identifying and reducing unplanned readmissions in the elderly, the risk of post-pneumonia readmissions in patients under the age of 65 years is largely unknown.[3–5] A few, hospital-[6–12] and state-level[13] studies have characterized re-hospitalizations in young adults with rates of readmission varying between 7.1% and 14.4%.[6, 8, 13] Furthermore, while prior studies have reported advanced age, high comorbidity burden, and unemployment as potential reasons increasing the risk of readmission in the elderly, patient demographic factors and comorbid illnesses influencing readmission risk in young and middle-aged adults are not known.[4, 8, 14] Moreover, since there are hospital and regional differences in patient composition and management of pneumonia, a comprehensive assessment of the burden of readmissions across all age groups is needed at a national-level to assess care priorities and potential targets for health policy interventions.

To address this, we sought to examine the burden of pneumonia readmissions across all age groups, and study patient characteristics that are associated with high readmission rates after an index hospitalization for pneumonia using the Nationwide Readmissions Database (NRD) for the years 2013 and 2014. We hypothesized that characteristics of patients readmitted after hospital discharge for pneumonia would vary with age and would be influenced by demographic and comorbidity characteristics.

## Methods

### Data source

We used the Agency of Healthcare Research and Quality’s (AHRQ’s) nationally representative, all-payer database, the Nationwide Readmissions Database (NRD) for the years 2013 and 2014. The database has been described previously.[15–17] Briefly, the NRD is designed as an annual dataset constructed from all hospitalizations captured in the State Inpatient Database (SID) of geographically-dispersed participating states, 21 in the year 2013 and 22 in 2014. For 2013 and 2014, the NRD captured 49.3% and 51.2% of the total U.S. resident population, and represented 49.1% and 49.3% respectively of all US hospitalizations during these years, respectively. In the NRD, all patients with one or more inpatient hospitalizations are assigned a verified de-identified patient linkage number allowing tracking of discharges for an individual across hospitals within a state throughout a calendar year. While State Inpatient Databases contain separate inpatient records of discharges and admissions that have the same date, in order to accurately capture readmissions, in NRD, these pairs of records are combined into one common record and ascribed to the institution where the final disposition occurred. Further, NRD is accompanied by discharge weights to generate national estimates. These discharge weights are generated by dividing the number of discharges nationally (from participating and non-participating states) by the number captured in the NRD within strata defined by patient (gender, and age groups: 0, 1–17, 18–44, 45–64, ≥65 years) and hospital (census region, urban/rural location, teaching status, size of the hospital defined by the number of beds, and hospital control) characteristics.

### Study cohort

We identified all adults aged ≥18 years who were hospitalized during 2013 and 2014 with a primary diagnosis of pneumonia using the International Classification of Diseases-9th Clinical Modification (ICD-9CM) codes for pneumonia (480.x, 481, 482.xx, 483.x, 485, 486, 487.0, and 488.11), as used by the Center of Medicare and Medicaid Services in reporting pneumonia readmission measures.[18] We included all observations with a principal diagnosis of pneumonia discharged alive between January 1, 2013—November 30, 2013 and January 1, 2014- November 30, 2014 allowing for a 30-day follow up period for each year. Patient discharged against medical advice were excluded. Since the NRD and its recommended analytic approach do not exclude index events occurring within 30-days from discharge from a preceding index event, in our analyses, a readmission event can serve as an index event itself.

### Study variables and outcomes

For each patient with an index event, we identified 30-day readmission as the first subsequent hospitalization for any cause within 30 days of discharge following a primary pneumonia hospitalization. We examined patient demographics (age and gender), insurance (Medicare, Medicaid, private, other, uninsured), income status (median household income based on zip code of residence, available as quartiles of income), admission status (elective or non-elective), hospital charges, length of stay and comorbidities.

To assess comorbidities as predictors of readmission, we used clinical classifications software (CCS) categories, a classification system which combines clinically meaningful ICD codes into 259 broad categories.[19] The CCS categories are substantively similar to clinically coherent condition categories (CCs) used by CMS in their risk-adjusted model for 30-day readmission.[18] These included asthma, chronic obstructive pulmonary disease, pleurisy, smoking, diabetes mellitus, hypertension, history of coronary artery bypass grafting (CABG), chronic coronary artery disease, acute myocardial infarction, valvular heart disease, cardiac arrhythmias, congestive heart failure, peripheral vascular disease, acute stroke or transient ischemic attack, hepatobiliary disease, anemia and other blood disorders, leukemia or metastatic malignancy, major solid cancers, disorders of fluid/ electrolyte/ acid base, chronic kidney disease, acute kidney injury, end-stage renal disease/need for hemodialysis, cardiorespiratory failure, protein calorie malnutrition, history of infection, chronic skin ulcer, paralysis, history of pneumonia, urinary tract infection, drug or alcohol use, sepsis, other gastrointestinal disorders, injuries, mood or anxiety disorders and other psychiatric disorders. Furthermore, since our study cohort spans across all age groups, we conducted a sensitivity analysis with the inclusion of 29 comorbidities developed by Elixhauser et al for use in all payer databases and report the potential predictors of readmissions from these models.

The primary outcome for the study was 30-day readmission, which was addressed for the overall population as well as for pre-defined age groups, 18–44 years, 45–64 years and ≥65 years of age.

### Statistical analysis

We compared baseline demographic characteristics and prevalence of comorbidities for patients within defined age groups of 18–44 years, 45–64 years and ≥65 years. We tested for differences across age-groups using the Scott-Chi square test for categorical variables and for the change in the distribution of continuous variables (age, length of stay and cost of hospitalization) across the three age groups using linear regression. These tests explicitly accounted for the clustering and stratification of data derived from a complex survey. Next, we obtained unadjusted estimates of 30-day readmission following an index hospitalization for pneumonia, both overall and for each of these age groups, expressed as percentage of all discharges. We used discharge weights provided in the NRD database to obtain national-level estimates of readmissions.[20]

In the analyses addressing predictors of readmission, we used a logistic regression model that accounted for clustering for patients at hospitals, and for the clustering and stratification of data. Our analyses were consistent with the approach outlined in a recent methodology paper by members of our group for the use of data from the AHRQ.[21, 22] In our risk-adjustment model, we included variables previously validated for pneumonia 30-day readmission in the Medicare population with comorbidities as detailed above. In addition to the above comorbidities, we adjusted for gender as well as income status and examined the interaction of gender and income status with the previously defined age groups to determine their effect on readmissions.

Further, to address whether predictors of readmission varied across age groups, we constructed additional models where we examined the interaction of age group (above or below 65 years of age) with the all covariates included in the model. For these analyses, in order to use the widest set of comorbidities that were not specific to any particular age group, we used Elixhauser comorbidities as the patient-level covariates included in the model. We also obtained estimates of hospitalization cost for both the index pneumonia hospitalization as well as re-hospitalization by multiplying the claim charge reported in NRD for each hospitalization with the cost-to-charge ratio for the respective hospitals.[23]

All patient-level analyses were performed in accordance with survey methodology recommended by HCUP.[20] All analyses were performed using SAS 9.4 (Cary, NC). Statistical significance was set as a two-tailed p-value ≤0.05. This project was reviewed by the Institutional Review Board of the University of Texas Southwestern Medical Center and was determined to be exempt from review.

## Results

We identified 629,939 patients who were discharged alive following an index hospitalization for pneumonia between January to November for the years 2013 and 2014 with a weighted estimate of 1,472,069 discharges nationally. With 532,481 estimated discharges, adults <65 years of age constituted more than one third of this cohort (36.1%). The overall mean age was 68.9 years and females represented more than 50% of discharges after an index stay for pneumonia in all three age groups (18–44 years, 45–64 years and ≥ 65 years). Cardiovascular comorbidities including coronary artery disease, history of CABG, arrhythmias, valvular heart disease and acute myocardial infarction were significantly more frequent in older adults. Most other comorbidities including COPD, tobacco smoking, malignancy, malnutrition and anemia were also more commonly noted in the elderly while asthma was more common in younger adults. Cardiorespiratory failure was more prevalent in older adults while sepsis was seen more frequently in younger adults. Medicare was the primary payer for 69.7% of index hospital stays overall and for 92.5% of patients ≥ 65 years of age. All patient characteristics are presented in Table 1. Characteristics were comparable in the years 2013 and 2014 with no significant differences (data not shown).

**Table 1:** Characteristics and comorbidities of patients discharged alive after an index hospitalization for pneumonia between 2013–14 in the National Readmissions Database sample, overall and by age- group.

Characteristic	Overall	18–44 years	45–64 years	>65 years	P-value
Number of index hospitalizations (weighted numbers ± SD)	1,472,069 (17761)	143,029 (2185)	389,452 (5039)	939,589 (12168)	
**Patient characteristics**					
Mean Age in years (SEM)	68.9 (0.1)	33.9 (0.1)	55.9 (0.0)	79.2 (0.0)	
Female Sex	53.0 (0.1)	52.9 (0.2)	51.5 (0.2)	53.7 (0.1)	<0.001
Income quartiles^§^					<0.001^|||^
0–25	31.2(0.5)	36.8 (0.6)	36.2 (0.6)	28.2 (0.5)	
26 to 50	28.9 (0.4)	28.0 (0.4)	28.5 (0.4)	29.2 (0.4)	
51 to 75	22.6 (0.4)	21.0 (0.4)	21.2 (0.4)	23.5 (0.4)	
76 to 100	17.3 (0.5)	14.1 (0.4)	14.1 (0.4)	19.1 (0.5)	
**Secondary diagnoses/comorbidities**					
Asthma	12.1 (0.1)	23.9 (0.2)	15.2 (0.2)	9.0 (0.1)	<0.001
COPD	36.7 (0.2)	10.5 (0.2)	36.1 (0.3)	40.9 (0.2)	<0.001
Smoking	18.4 (0.2)	7.4 (0.1)	15.8 (0.2)	21.3 (0.3)	<0.001
Diabetes Mellitus	32.9 (0.1)	18.7 (0.2)	34.9 (0.2)	34.3 (0.1)	<0.001
Hypertension	63.4 (0.2)	27.6 (0.3)	56.5 (0.2)	71.8 (0.2)	<0.001
Acute myocardial infarction	1.2 (0.0)	0.2 (0.0)	0.7 (0.0)	1.5 (0.0)	<0.001
Coronary artery disease	26.2 (0.2)	3.2 (0.1)	17.0 (0.1)	33.6 (0.2)	<0.001
Prior CABG	6.1 (0.1)	0.3 (0.0)	3.0 (0.0)	8.3 (0.1)	<0.001
TIA/ CVA	0.5 (0.0)	0.1 (0.0)	0.3 (0.0)	0.7 (0.0)	<0.001
Valvular heart disease	7.8 (0.1)	2.5 (0.1)	4.0 (0.1)	10.2 (0.1)	<0.001
Cardiac arrhythmia	26.2 (0.2)	10.9 (0.2)	14.8 (0.2)	33.6 (0.2)	<0.001
Hepatobiliary disease	5.9 (0.1)	6.2 (0.1)	7.7 (0.1)	5.1 (0.1)	<0.001
Anemia	28.3 (0.2)	23.4 (0.3)	25.5 (0.2)	30.2 (0.2)	<0.001
Solid Malignancy	18.3 (0.1)	4.7 (0.1)	15.0 (0.2)	21.7 (0.1)	<0.001
Leukemia/Metastatic malignancy	4.6 (0.1)	2.2 (0.1)	5.1 (0.1)	4.7 (0.1)	<0.001
Acute Kidney Injury	13.0 (0.1)	5.8 (0.1)	10.3 (0.1)	15.1 (0.1)	<0.001
End Stage Renal Disease/ Hemodialysis	4.2 (0.1)	4.6 (0.1)	5.7 (0.1)	3.6 (0.0)	<0.001
Chronic kidney disease	15.7 (0.1)	3.2 (0.1)	8.6 (0.1)	20.5 (0.2)	<0.001
Fluid/electrolyte disorder	36.7 (0.2)	32.2 (0.3)	35.8 (0.2)	37.8 (0.2)	<0.001
Paralysis	1.5 (0.0)	4.5 (0.1)	2.1 (0.0)	0.8 (0.0)	<0.001
Chronic skin ulcer	3.7 (0.0)	1.7 (0.1)	2.8 (0.0)	4.3 (0.0)	<0.001
Dementia/ Delirium	12.6 (0.1)	0.4 (0.0)	1.8 (0.0)	18.9 (0.1)	<0.001
Malnutrition	9.1 (0.1)	5.6 (0.2)	7.8 (0.1)	10.2 (0.1)	<0.001
Alcohol/Drug use disorder	5.9 (0.1)	12.7 (0.2)	11.7 (0.2)	2.4 (0.0)	<0.001
Mood/anxiety disorder	23.9 (0.2)	25.2 (0.3)	30.0 (0.2)	21.2 (0.2)	<0.001
Psychotic disorders	8.7 (0.1)	4.7 (0.1)	5.3 (0.1)	10.7 (0.1)	<0.001
**Complications**					
Sepsis	3.2 (0.0)	3.9 (0.1)	3.6 (0.1)	3.0 (0.0)	<0.001
Cardiac arrest	0.2 (0.0)	0.1 (0.0)	0.2 (0.0)	0.2 (0.0)	0.049
Respiratory failure	25.3 (0.3)	17.0 (0.3)	25.3 (0.3)	26.6 (0.3)	<0.001
Cardiorespiratory failure	26.4 (0.3)	18.1 (0.3)	26.6 (0.3)	27.6 (0.3)	<0.001
Shock	0.5 (0.0)	0.5 (0.0)	0.6 (0.0)	0.5 (0.0)	<0.001
**Procedures**					
Mechanical ventilation	6.3 (0.1)	5.8 (0.2)	7.4 (0.1)	5.9 (0.1)	<0.001
ECMO	0.0 (0.0)	0.04 (0.0)	0.0 (0.0)	0.0 (0.0)	<0.001
**Administrative/ financial details**					
Weekend admission	25.3 (0.1)	25.5 (0.2)	25.0 (0.1)	25.3 (0.1)	0.222
Payment source					<0.001*
Medicare	69.7 (0.2)	16.6 (0.2)	34.3 (0.2)	92.5 (0.2)	
Medicaid	9.2 (0.1)	30.4 (0.3)	20.8 (0.2)	1.2 (0.0)	
Private insurance	14.9 (0.2)	33.2 (0.4)	32.4 (0.3)	4.9 (0.1)	
Others	6.1 (0.1)	19.8 (0.3)	12.5 (0.2)	1.4 (0.1)	

SD—standard deviation, SEM—standard error of mean, C.I.—confidence interval. COPD—Chronic obstructive pulmonary disease, CAD—Coronary artery disease, CABG—coronary artery bypass grafting, TIA—transient ischemic attack, CVA -Cerebrovascular accident, ECMO—Extracorporeal membrane oxygenation. All numbers represent percentages (standard errors), unless otherwise specified.

§Median household income quartiles based on patient zip code

||trend for ‘lowest quartile (0-25^th^ percentile)’ vs ‘others’

*trend for Medicare vs others. Reporting of elective status may vary by states.

For further description of data elements, readers are encouraged to consult the AHRQ website at ‘https://www.hcup-us.ahrq.gov/db/nation/nrd/nrddde.jsp’.

The unadjusted rate of 30-day readmission was 16.2% for the entire cohort. Readmission rate was highest in the elderly at 16.7%, substantial in the middle age group at 16.1% and lowest in young adults at 12.4% (p-value for trend <0.0001). Compared to women, men were more likely to be readmitted (OR 1.05, 95% CI 1.03–1.07) overall. After adjustment for comorbidities, middle-aged adults had small but statistically significantly higher odds of readmission compared to the elderly (risk adjusted OR 1.04, 95% CI 1.01–1.07). Patients in the lowest income quartile were also more likely to be readmitted than those in the highest income quartile (risk adjusted OR 1.09, 95% CI 1.04–1.13).

**Fig 1:**
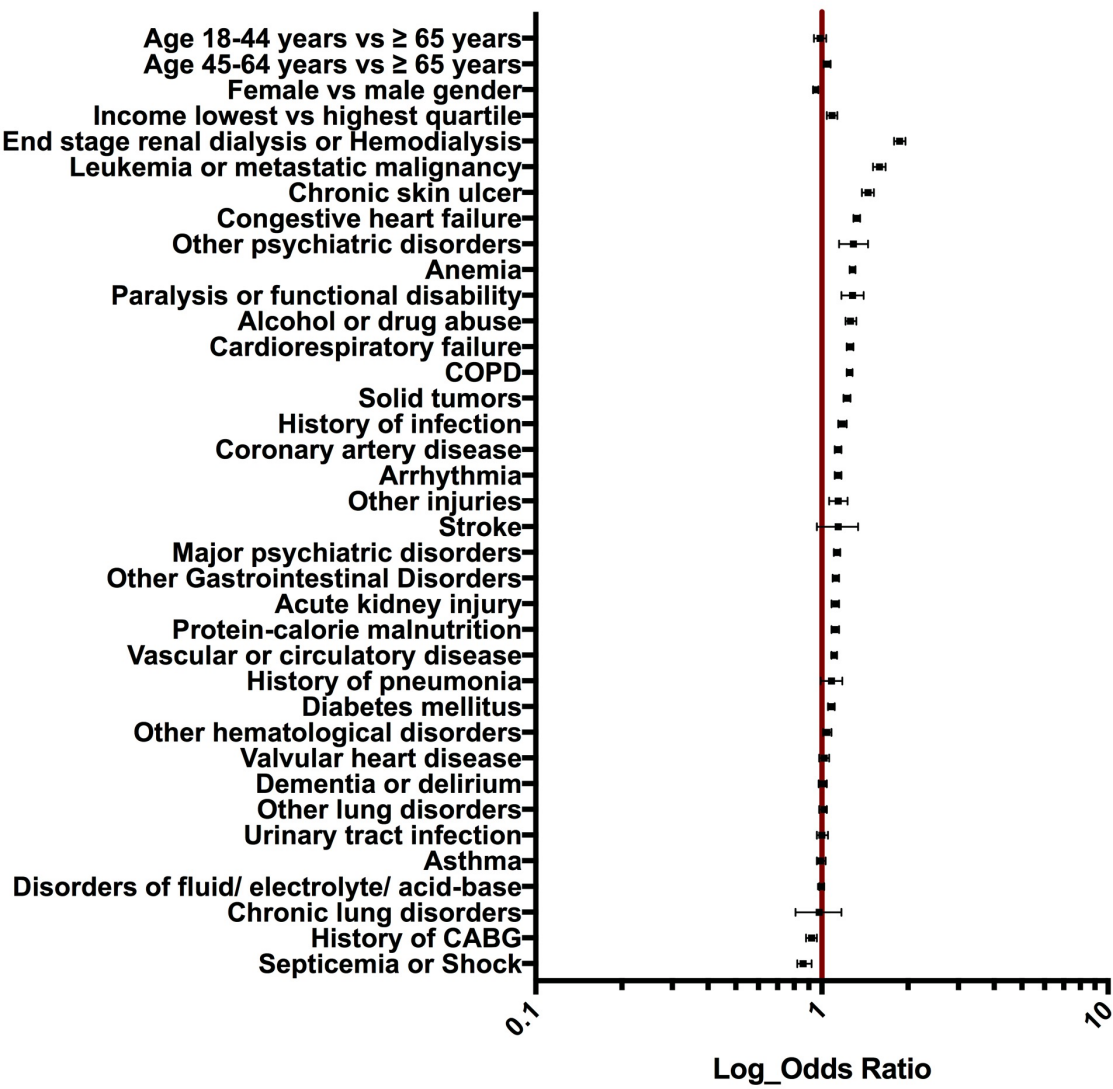
Risk-adjusted odds ratios for readmission by age, gender, income and comorbidities.

The current CMS risk-adjustment model for comorbidities performed modestly in our all-payer population, which included patients below 65 years of age (model c-statistic 0.63). Among comorbid conditions, end stage renal disease or being on dialysis was associated with the highest odds of re-hospitalization (risk adjusted OR 1.88, 95% CI 1.79–1.96). Other comorbid conditions including metastatic malignancy or leukemia (risk adjusted OR 1.59, 95% CI 1.57–1.61), chronic skin ulcer (risk adjusted OR 1.45, 95% CI 1.38–1.52), congestive heart failure (risk adjusted OR 1.33, 95% C.I 1.29–1.36) and anemia (risk adjusted OR 1.28, 95% C.I 1.25–1.31) were also significant predictors of 30-day pneumonia readmission. A forest plot of predictors affecting risk of readmission is shown in Fig 1 and odds ratios are detailed in S1 Table.

In the interaction analysis, after adjustment for comorbidities and income, men in middle (risk adjusted OR 1.1, 95% CI 1.06, 1.15) and older age groups (risk adjusted OR 1.03, 95% CI 1.01, 1.06) had higher odds of being readmitted than women in the same age categories (P for age*gender interaction 0.020). There was no significant interaction between age or gender and income quartile on readmission risk.

In sensitivity analyses, we used the 29 prognostically relevant comorbidities defined by Elixhauser et al in a logistic regression model (S2 Table). We found that among comorbidities that are not included in the CMS model but are defined as Elixhauser comorbidities, collagen vascular diseases, liver disease, obesity, weight loss and pulmonary circulation diseases were significant predictors of readmission. Hypertension, acquired immune deficiency syndrome and hypothyroidism were also included as Elixhauser comorbidities but were not found to be significant predictors of readmission in this cohort. Moreover, in our assessment of the interaction between sex and three age groups (18–44, 45–64, and >65 years), results from the model based on Elixhauser comorbidities were similar to those based on the CMS model.

Notably, in both the CMS model and the one based on Elixhauser’s comorbidities, males had a higher risk-adjusted odds for readmission compared to females in middle aged (OR 1.12, 95% C.I. 1.09–1.15 in CMS model versus OR 1.10, 95% C.I. 1.06–1.15 in the one with Elixhauser’s comorbidities) and elderly patients with pneumonia (OR 1.05, 95% C.I. 1.03–1.07 in CMS versus OR 1.03, 95% C.I. 1.01–1.06 in the one with Elixhauser’s comorbidities) in these age groups when adjusted for the Elixhauser comorbidities.

Further, we assessed the differences in predictors for readmissions across patients aged 65 years and older compared with those less than 65 years of age. Using Elixhauser comorbidities in the risk adjustment model, we found a significant interaction by age group (S3 Table). However, for most comorbid conditions, the odds of readmission risk varied in the same direction for both young and elderly patients with a difference predominantly in the magnitude of effect sizes. This was most prominent for solid tumors, lymphoma, metastatic cancers and renal failure with these comorbidities predicting a higher risk of readmission in patients less than 65 years of age compared to those 65 years of age or older. Notably, hypertension was independently associated with higher odds for readmission risk in patients less than 65 years of age whereas it was associated with lower readmission odds in those 65 years and older (risk adjusted OR: 1.06, 95% C.I. 1.03–1.09 versus risk adjusted OR: 0.97, 95% C.I. 0.95–0.99, P for interaction, <0.001).

Overall cost of index hospitalizations was $14.8 billion with mean cost per hospital stay of $9,305.9 for the 18–44 year age group, $10,238.7 for 45–64 year age group and $10,163.9 for patients ≥ 65 years (Table 2). Overall cost of readmissions was estimated at $3.5 billion for the entire cohort. Notably, mean cost per hospital stay was significantly higher for middle-aged adults at $15,976.3 compared to young adults ($15,539.2) and the elderly ($14,442.7).

**Table 2:** Outcomes of patients discharged alive after an index hospital stay for pneumonia in the National Readmissions Sample 2013–2014, overall and by age groups. All numbers are percentages with SE in parenthesis unless specified otherwise.

Characteristics	Overall	18–44 years	45–64 years	≥ 65 years	P value
**Index hospitalization**					
Length of stay, days (mean ± SEM)	5.2 (0.0)	4.5 (0.0)	5.0 (0.0)	5.4 (0.0)	<0.001
Cost of hospitalization (US$)	10,103	9,306	10,239	10,164	<0.001
Disposition					<0.001
Home or self-care	58.5 (0.2)	86.3 (0.2)	76.3 (0.2)	47.0 (0.2)	
Short term hospital	1.0 (0.0)	0.9 (0.0)	1.0 (0.0)	1.0 (0.0)	
Skilled care facility	21.9 (0.1)	3.3 (0.1)	9.1 (0.1)	30.0 (0.2)	
Home health care	17.3 (0.2)	6.0 (0.2)	11.4 (0.2)	21.4 (0.2)	
Others	1.2 (0.0)	3.6 (0.1)	2.2 (0.0)	0.5 (0.0)	
**30-day Readmission**					
Unadjusted all-cause rate, %	16.2%	12.4%	16.1%	16.7%	<0.001
Length of stay of index hospitalization in those readmitted, days (mean ± SEM)	6.2 (0.0)	5.7 (0.0)	6.0 (0.0)	6.4 (0.0)	<0.001
Cost of hospitalization (US$)	14,919	15,539	15,976	14,443	<0.001

## Discussion

In our study assessing 30-day pneumonia readmission in a nationally representative all-payer sample of hospital discharges in the US, we made the following key observations. First, although the risk of readmission after a hospital discharge for pneumonia is highest for the elderly, it is substantial for middle-aged adults. This risk remains high after adjusting for comorbidities. Second, men were at a higher risk of being readmitted compared to women in the overall population, and on specific gender and age interaction analysis, this risk was found to be significant for the middle and elderly age groups. Third, we identified multiple comorbidities, most prominently, end stage renal disease, that increase the risk of patients being readmitted after discharge even after adjusting for age. Finally, we found that readmissions pose a substantial economic burden, with a higher cost of readmission for the middle-aged population per hospital stay compared to both young adults and the elderly.

With the largest number of hospitalizations for a medical diagnosis, pneumonia accounts for extensive hospital costs and preventing readmissions is a potential avenue to mitigate this resource use.[1] However, most data regarding the burden of pneumonia readmissions is derived from Medicare patients with analysis of readmission patterns in younger age groups limited to individual hospital and state level studies. To our knowledge, this is the first description of readmission patterns following pneumonia in a nationally representative sample in the United States that includes patients from all ages and all payers across a span of two years.

Our study elucidates that although the risk of readmission increases with advancing age, the burden of readmission among middle-aged adults is substantial. Krumholz et al reported similar results from the California State Inpatient Database and confirmation of this burden at a national level is an important finding that deserves consideration.[13] Recognition of the significant number of middle-aged adults who are readmitted after hospital discharge for pneumonia is the first step at identifying them as an “at risk” group prompting further investigation into factors that influence it and strategies to mitigate it.

The impact of gender on outcomes following a hospitalization for pneumonia is less well studied. Mongardon et al described increased mortality in males admitted to the ICU with severe pneumococcal pneumonia whereas Caceres et al did not find gender differences in a similar setting. [24, 25] In a prospective cohort study of adults older than 65 years, males were found to be at higher risk of readmission after hospitalization for community acquired pneumonia.[14] We found males in middle and older age groups to be at a slightly higher risk of readmission compared to females. Further studies focusing on severity of illness, adherence to treatment, and compliance with follow up are needed to understand the reasons for these gender differences in readmission risk.

Similar to readmission after discharge for acute myocardial infarction and heart failure, readmission after pneumonia has also been found to be associated with comorbid illnesses in prior studies.[3, 6, 8] We found that patients with end stage renal disease had twice the odds of being re-hospitalized even after adjusting for age and other comorbidities. Prior studies have noted chronic kidney disease or the need for renal replacement therapy to be independent predictors of both re-hospitalization and mortality. [2, 7, 24, 26] Possible reasons for this could include special considerations regarding antibiotic dosing in patients with poor kidney function, underlying immune dysfunction and dialysis needs.[27]

Another significant finding in our study is the substantial cost burden of pneumonia re-hospitalization and the strikingly higher cost per hospital stay for middle-aged adults compared to both young and elderly patients. With 16.1% of adults in this age group being readmitted following discharge from a hospital stay for pneumonia, the cost burden of readmissions is significant enough to warrant attention. Reasons for this are unclear however a possible factor could be severity of illness. CURB-65, one of the most commonly used admission criteria for pneumonia by emergency departments is more readily met by definition in patients older than 65 years whereas younger patients must exhibit at least two features such as confusion, uremia, tachypnea or hypotension marking a higher severity of illness. The NRD does not allow an assessment of disease severity, therefore, further studies are needed to evaluate this and assess if this population warrants closer attention at discharge with regards to in-hospital functional decline, medication adherence and follow up visits to prevent readmission.

Our study has a few limitations. First, data on readmissions were only available for the years 2013 and 2014 at the time of analysis. It is, therefore, unknown if these findings are consistent over time. Second, NRD is constructed from State Inpatient Databases and therefore readmissions occurring in another state are not captured. However in large sensitivity analysis reported by AHRQ less than 5% of readmission estimates are affected by readmissions across state borders.[28] Third, information on race and ethnicity is not available and therefore the impact of these on readmissions cannot be assessed. Fourth, given the design of the NRD, we were unable to adequately pursue hospital-level analyses or those that span multiple years. Fifth, although conceptually similar, our risk adjustment model is not the same as that adopted by CMS for pneumonia readmission since we used CCS codes rather than CCs in accordance with the AHRQ recommendations for NRD. However, this approach has been used in prior studies addressing readmissions for other conditions[29] and the performance of the CMS model for risk-adjustment used by national policymakers was comparable in our study (c-statistic 0.63) to that in the original fee-for-service Medicare population (c-statistic 0.63). Furthermore, we performed a sensitivity analysis using the 29 comorbidities developed by Elixhauser et al specifically for use in all payer databases and found that potential predictors of readmissions were comparable in these models. Finally, diagnosis of pneumonia and comorbidities used in the risk-adjustment model are derived from administrative claims codes, and important clinical information on disease severity and therapeutic strategies is not available. However, these are known challenges in working with administrative data. [30] We therefore relied on the comorbidities identified in prior models for identifying predictors of readmission risk.

In conclusion, our results highlight the burden of 30-day readmission in young and middle-aged pneumonia survivors and underscore the need for further studies focusing on this population to understand gender, disease severity, comorbidity and treatment related factors that may influence this risk.

## Supporting information

S1 Table Risk- adjusted odds ratios for readmission within 30 days after discharge following a hospitalization for pneumonia using the risk adjustment model adopted by Centers for Medicare and Medicaid Services. (DOCX)

S2 Table Risk- adjusted odds ratios for readmission within 30 days after discharge following a hospitalization for pneumonia using the Elixhauser model. (DOCX)

S3 Table Risk- adjusted odds ratios for readmission within 30 days after discharge following a hospitalization for pneumonia using the Elixhauser model in patients less than 65 years of age versus those 65 and older. P value presented for interaction analysis between age and each characteristic.
(DOCX)
