# Supplementary material for: Readmissions of adults within three age groups following hospitalization for pneumonia: Analysis from the Nationwide Readmissions Database
Source: PLoS One. 2018 Sep 13;13(9):e0203375. doi: 10.1371/journal.pone.0203375 (PMC6136736; doi:10.1371/journal.pone.0203375)
Supplement: S1 Table — (DOCX) [file pone.0203375.s001.docx]

Online Supplement

**Readmissions in Adults Following Hospitalization for Pneumonia Across Age Groups: Analysis from the Nationwide Readmissions Database**

Snigdha Jain, MD; Rohan Khera, MD; Eric M Mortensen, MD, MSc; Jonathan Weissler, MD

S1 Table. Risk- adjusted odds ratios for readmission within 30 days after discharge following a hospitalization for pneumonia using the risk adjustment model adopted by CMS

| Characteristic | Odds ratio  (95% Confidence Intervals) |
| --- | --- |
| Age 18-44 years vs ≥ 65 years | 0.99 (0.94 - 1.04) |
| Age 45-64 years vs ≥ 65 years | 1.04 (1.01 - 1.07) |
| Female vs male gender | 0.95 (0.93 - 0.97) |
| Income lowest vs highest quartile | 1.09 (1.04 - 1.13) |
| End stage renal disease or hemodialysis | 1.87 (1.79 - 1.96) |
| Leukemia or metastatic malignancy | 1.59 (1.51 - 1.67) |
| Chronic skin ulcer | 1.45 (1.38 - 1.52) |
| Congestive heart failure | 1.33 (1.29 - 1.36) |
| Other psychiatric disorders | 1.29 (1.15 - 1.45) |
| Anemia | 1.28 (1.25 - 1.31) |
| Paralysis or functional disability | 1.28 (1.17 - 1.40) |
| Alcohol or drug abuse | 1.26 (1.21 - 1.32) |
| Cardiorespiratory failure | 1.25 (1.22 - 1.29) |
| COPD | 1.25 (1.22 - 1.28) |
| Solid tumors | 1.22 (1.19 - 1.26) |
| History of infection | 1.18 (1.14 - 1.22) |
| Coronary artery disease | 1.14 (1.11 - 1.17) |
| Arrhythmias | 1.14 (1.11 - 1.17) |
| Other injuries | 1.14 (1.06 - 1.23) |
| Stroke | 1.14 (0.96 - 1.34) |
| Major psychiatric disorders | 1.13 (1.10 - 1.16) |
| Other gastrointestinal disorders | 1.12 (1.09 - 1.15) |
| Acute kidney injury | 1.12 (1.08 - 1.15) |
| Protein calorie malnutrition | 1.11 (1.08 - 1.15) |
| Vascular or circulatory disease | 1.10 (1.08 - 1.13) |
| History of pneumonia | 1.08 (0.99 - 1.18) |
| Diabetes mellitus | 1.08 (1.05 - 1.11) |
| Other hematological disorders | 1.04 (1.01 - 1.08) |
| Valvular heart disease | 1.02 (0.98 - 1.06) |
| Dementia or delirium | 1.01 (0.97 - 1.04) |
| Other lung disorders | 1.02 (0.98 - 1.04) |
| Urinary tract infection | 1.00 (0.96 - 1.05) |
| Asthma | 0.99 (0.96 - 1.03) |
| Disorders of fluid/ electrolyte/ acid-base | 0.99 (0.98 - 1.02) |
| Chronic lung disorders | 0.98 (0.81 - 1.17) |
| History of CABG | 0.92 (0.88 - 0.96) |
| Septicemia or shock | 0.86 (0.82 - 0.92) |
