# Supplementary material for: Readmissions of adults within three age groups following hospitalization for pneumonia: Analysis from the Nationwide Readmissions Database
Source: PLoS One. 2018 Sep 13;13(9):e0203375. doi: 10.1371/journal.pone.0203375 (PMC6136736; doi:10.1371/journal.pone.0203375)
Supplement: S2 Table — (DOCX) [file pone.0203375.s002.docx]

Online Supplement

**Readmissions in Adults Following Hospitalization for Pneumonia Across Age Groups: Analysis from the Nationwide Readmissions Database**

Snigdha Jain, MD; Rohan Khera, MD; Eric M Mortensen, MD, MSc; Jonathan Weissler, MD

S2 Table. Risk- adjusted odds ratios for readmission within 30 days after discharge following a hospitalization for pneumonia using the Elixhauser model

| Characteristic | Odds ratio  (95% Confidence Intervals) |
| --- | --- |
| Age 18-44 years vs ≥ 65 years | 0.92 (0.88 - 0.97) |
| Age 45-64 years vs ≥ 65 years | 1.06 (1.03 - 1.09) |
| Female vs male gender | 0.96 (0.94 - 0.97) |
| Income lowest vs highest quartile | 1.09 (1.06 - 1.12) |
| Congestive Heart Failure | 1.44 (1.41 - 1.47) |
| Valvular disease | 1.04 (1.02 - 1.07) |
| Hypertension | 1.02 (1.00 - 1.04) |
| Diabetes with chronic complications | 1.24 (1.20 - 1.29) |
| Diabetes without chronic complications | 1.11 (1.09 - 1.13) |
| Acquired Immune Deficiency Syndrome | 1.02 (0.70 - 1.49) |
| Coagulopathy | 1.12 (1.08 - 1.16) |
| Chronic blood loss anemia | 1.27 (1.15 - 1.39) |
| Deficiency anemias | 1.27 (1.24 - 1.29) |
| Pulmonary circulation disease | 1.20 (1.17 - 1.24) |
| Paralysis | 1.39 (1.33 - 1.45) |
| Hypothyroidism | 1.03 (1.00 - 1.05) |
| Lymphoma | 1.47 (1.39 - 1.55) |
| Weight loss | 1.24 (1.20 - 1.27) |
| Obesity | 0.91 (0.89 - 0.94) |
| Peripheral vascular disease | 1.15 (1.11 - 1.18) |
| Other neurological disorders | 1.13 (1.10 - 1.16) |
| Renal failure | 1.35 (1.32 - 1.38) |
| Solid tumor without metastasis | 1.64 (1.58 - 1.70) |
| Metastatic cancer | 2.07 (1.99 - 2.15) |
| Alcohol abuse | 1.14 (1.09 - 1.19) |
| Drug abuse | 1.29 (1.23 - 1.35) |
| Chronic pulmonary disease | 1.26 (1.24 - 1.29) |
| Liver disease | 1.26 (1.21 - 1.32) |
| Fluid and electrolyte disorders | 1.04 (1.02 - 1.05) |
| Psychoses | 1.29 (1.25 - 1.33) |
| Peptic ulcer disease | 0.88 (0.58 - 1.34) |
| Rheumatoid arthritis or collagen vascular disease | 1.16 (1.12 - 1.21) |
| Depression | 1.09 (1.07 - 1.11) |
