# Supplementary material for: Readmissions of adults within three age groups following hospitalization for pneumonia: Analysis from the Nationwide Readmissions Database
Source: PLoS One. 2018 Sep 13;13(9):e0203375. doi: 10.1371/journal.pone.0203375 (PMC6136736; doi:10.1371/journal.pone.0203375)
Supplement: S3 Table — P value presented for interaction analysis between age and each characteristic. (DOCX) [file pone.0203375.s003.docx]

Online Supplement

**Readmissions in Adults Following Hospitalization for Pneumonia Across Age Groups: Analysis from the Nationwide Readmissions Database**

Snigdha Jain, MD; Rohan Khera, MD; Eric M Mortensen, MD, MSc; Jonathan Weissler, MD

S3 Table. Risk- adjusted odds ratios for readmission within 30 days after discharge following a hospitalization for pneumonia using the Elixhauser model in patients less than 65 years of age versus those 65 and older. P value presented for interaction analysis between age and each characteristic.

| Characteristic | Odds Ratio  (95% Confidence Interval) | P value for interaction effect |
| --- | --- | --- |
| Female vs male gender |  | <0.001 |
| < 65 years | 0.92 (0.89 - 0.94) |  |
| ≥ 65 years | 0.95 (0.93 - 0.96) |  |
| Income lowest vs highest quartile |  | <0.001 |
| < 65 years | 1.11 (1.06 - 1.17) |  |
| ≥ 65 years | 1.08 (1.04 - 1.12) |  |
| Congestive Heart Failure |  | <0.001 |
| < 65 years | 1.64 (1.57 - 1.70) |  |
| ≥ 65 years | 1.37 (1.34 - 1.40) |  |
| Valvular disease |  | 0.003 |
| < 65 years | 1.10 (1.02 - 1.18) |  |
| ≥ 65 years | 1.04 (1.00 - 1.07) |  |
| Hypertension |  | <0.001 |
| < 65 years | 1.06 (1.03 - 1.09) |  |
| ≥ 65 years | 0.97 (0.95 - 0.99) |  |
| Diabetes with chronic complications |  | <0.001 |
| < 65 years | 1.30 (1.22 - 1.38) |  |
| ≥ 65 years | 1.17 (1.13 - 1.22) |  |
| Diabetes without chronic complications |  | <0.001 |
| < 65 years | 1.14 (1.11 - 1.18) |  |
| ≥ 65 years | 1.10 (1.07 - 1.12) |  |
| Acquired Immune Deficiency Syndrome |  | 0.991 |
| < 65 years | 1.00 (0.66 - 1.53) |  |
| ≥ 65 years | 1.09 (0.30 - 4.02) |  |
| Coagulopathy |  | <0.001 |
| < 65 years | 1.19 (1.13 - 1.26) |  |
| ≥ 65 years | 1.08 (1.04 - 1.12) |  |
| Chronic blood loss anemia |  | <0.001 |
| < 65 years | 1.31 (1.09 - 1.57) |  |
| ≥ 65 years | 1.27 (1.13 - 1.43) |  |
| Deficiency anemias |  | <0.001 |
| < 65 years | 1.31 (1.27 - 1.35) |  |
| ≥ 65 years | 1.23 (1.21 - 1.26) |  |
| Pulmonary circulation disease |  | <0.001 |
| < 65 years | 1.26 (1.19 - 1.34) |  |
| ≥ 65 years | 1.17 (1.13 - 1.21) |  |
| Paralysis |  | <0.001 |
| < 65 years | 1.57 (1.46 - 1.68) |  |
| ≥ 65 years | 1.26 (1.19 - 1.33) |  |
| Hypothyroidism |  | 0.082 |
| < 65 years | 1.04 (0.99 - 1.09) |  |
| ≥ 65 years | 1.02 (0.99 - 1.04) |  |
| Lymphoma |  | <0.001 |
| < 65 years | 1.94 (1.76 - 2.14) |  |
| ≥ 65 years | 1.30 (1.22 - 1.39) |  |
| Weight loss |  | <0.001 |
| < 65 years | 1.32 (1.25 - 1.40) |  |
| ≥ 65 years | 1.20 (1.16 - 1.24) |  |
| Obesity |  | <0.001 |
| < 65 years | 0.87 (0.83 - 0.90) |  |
| ≥ 65 years | 0.96 (0.93 - 1.00) |  |
| Peripheral vascular disease |  | <0.001 |
| < 65 years | 1.19 (1.1 - 1.28) |  |
| ≥ 65 years | 1.14 (1.11 - 1.18) |  |
| Other neurological disorders |  | <0.001 |
| < 65 years | 1.33 (1.27 - 1.4) |  |
| ≥ 65 years | 1.05 (1.02 - 1.07) |  |
| Renal failure |  | <0.001 |
| < 65 years | 1.70 (1.63 - 1.77) |  |
| ≥ 65 years | 1.25 (1.22 - 1.29) |  |
| Solid tumor without metastasis |  | <0.001 |
| < 65 years | 2.37 (2.21 - 2.54) |  |
| ≥ 65 years | 1.42 (1.37 - 1.48) |  |
| Metastatic cancer |  | <0.001 |
| < 65 years | 2.94 (2.77 - 3.12) |  |
| ≥ 65 years | 1.69 (1.61 - 1.78) |  |
| Alcohol abuse |  | <0.001 |
| < 65 years | 1.23 (1.17 - 1.31) |  |
| ≥ 65 years | 1.06 (0.98 - 1.14) |  |
| Drug abuse |  | <0.001 |
| < 65 years | 1.30 (1.23 - 1.37) |  |
| ≥ 65 years | 1.29 (1.17 - 1.42) |  |
| Chronic pulmonary disease |  | <0.001 |
| < 65 years | 1.25 (1.22 - 1.29) |  |
| ≥ 65 years | 1.28 (1.25 - 1.30) |  |
| Liver disease |  | <0.001 |
| < 65 years | 1.31 (1.24 - 1.39) |  |
| ≥ 65 years | 1.16 (1.09 - 1.25) |  |
| Fluid and electrolyte disorders |  | <0.001 |
| < 65 years | 0.98 (0.96 - 1.01) |  |
| ≥ 65 years | 1.06 (1.04 - 1.08) |  |
| Peptic ulcer disease |  | 0.638 |
| < 65 years | 1.04 (0.54 - 2.02) |  |
| ≥ 65 years | 0.76 (0.43 - 1.35) |  |
| Rheumatoid arthritis or collagen vascular disease |  | <0.001 |
| < 65 years | 1.24 (1.16 - 1.32) |  |
| ≥ 65 years | 1.12 (1.06 - 1.17) |  |
| Psychoses |  | <0.001 |
| < 65 years | 1.44 (1.37 - 1.51) |  |
| ≥ 65 years | 1.18 (1.12 - 1.24) |  |
| Depression |  | <0.001 |
| < 65 years | 1.18 (1.14 - 1.23) |  |
| ≥ 65 years | 1.04 (1.01 - 1.07) |  |
